# Prevalence of vitamin D deficiency: cross-sectional study of a hospital cohort of HIV-1 infected outpatients

**DOI:** 10.1186/1758-2652-13-S4-P101

**Published:** 2010-11-08

**Authors:** M Cervero, V Alcázar, C García-La Calle, R Sanz, JL Agud

**Affiliations:** 1 H. Severo Ochoa, Internal Medicine, Leganés, Spain; 2 H. Severo Ochoa, Endocrinology Service, Leganés, Spain; 3 H. Severo Ochoa, Laboratory Service, Leganés, Spain

## Background

To examine the prevalence and causal factors of vitamin D deficiency among HIV-1 infected patients and specially to assess whether antiretroviral drugs interfere with vitamin D metabolism.

## Methods

We performed a cross-sectional study of a hospital cohort (n=147) of HIV-1 infected outpatients followed-up by the same physician. Data were collected between January 2008 and January 2010. We draw samples for the measurement of 25(OH)D3, PTHi, serum calcium, serum phosphate, alkaline phosphatase, CD4+ cell count and viral load. Data on age, sex, VIH infection risk group, weight, height, skin color, time since HIV diagnosis, duration and stage of the infection (according to CDC 1983 staging system), as well as duration of typo of antiretroviral therapy (ART). A nutrition specialist of the same hospital performed a survey of sunlight exposition and daily vitamin D intake. We performed bi- and multivariant analysis to identify risk factor related to vitamin D deficiency (25(OH)D l<20 µg/L).

## Results

Median age was 45 years; 67.3% were males and 89.1% Caucasians. CD4+ count was < 200 cells/µL in 15.6 %, and 76.2% had a viral load below 30 copies/ml. Median serum 25(OH)D level was 21.1 µg/L (IQR 12,8-28,3) and 47.6% had 25(OH)D < 20 µg/L. Multivariate analysis of predisposing factor to vitamin D deficiency showed decreasing risk in summer(OR 0.131, 95% CI 0.05-0.336, p=0.0001) and fall (OR 0.021 95% CI 0.005-0.089, p=0.0001) and increased risk in heterosexual (OR 2.77, 95% CI 1.06-7.21, p=0.036) and with the tenofovir use (OR 2.71, 95% CI 1.14-6.44, p=0.024). In univariate analysis, current nevirapine use was protective against development of vitamin D deficiency (OR 0.42, 95% CI 0.15-0.95, p=0.039). Black race was not a risk factor, but it was underrepresented in our sample.

## Conclusions

Despite the low latitude of Spain, moderate vitamin D deficiency in HIV infected patients is more prevalent in our cohort than in the cohorts of Switzerland, Netherlands and Boston. It was related to the season, heterosexual risk group and tenofovir use. Nevirapine use was associated with less risk in univariate analysis.

